# 

**Published:** 1883-01

**Authors:** 


					﻿0
ESTABLISHED 11ST 1855.
Old Series.	TAlVrTTAT?V IftftQ	New Serie ,
Vol. XX-No. 10.	JAAUAKI, 1OOO.	Vol. H-No.f
ATLANTA
MEDICAL REGISTER.
(ATLANTA MEDICAL AND SURGICAL JOURNAL.)
ZZITET) SB'S"
JAMES B. BAIRD, M.D.
H. H. DICKSON. PUBLISHER. TERMS, $2.50 A YEAR IN ADVANCE.
/
ATLANTA, GEORGIA:
B. B. DICKSON, BOOK AND JOB PRINTER AND BOOKBINDER.
1S83.
				

